# Fascial plane blocks for cardiothoracic surgery: a narrative review

**DOI:** 10.1186/s44158-024-00155-5

**Published:** 2024-03-11

**Authors:** Paolo Capuano, Giuseppe Sepolvere, Antonio Toscano, Paolo Scimia, Simona Silvetti, Mario Tedesco, Luca Gentili, Gennaro Martucci, Gaetano Burgio

**Affiliations:** 1 Department of Anesthesia and Intensive Care, Istituto Mediterraneo per i Trapianti e Terapie ad alta Specializzazione (IRCCS-ISMETT), UPMCI (University of Pittsburgh Medical Center Italy), Palermo, Italy; 2 Department of Anesthesia and Cardiac Surgery Intensive Care Unit, Casa Di Cura San Michele, Maddaloni, Caserta, Italy; 3 Department of Anesthesia, Critical Care and Emergency, “Città Della Salute E Della Scienza” Hospital, Turin, Italy; 4 Intensive Care Unit, Department of Anesthesia, G. Mazzini Hospital, Teramo, Italy; 5 Department of Cardioanesthesia and Intensive Care, Policlinico San Martino IRCCS Hospital – IRCCS Cardiovascular Network, Genoa, Italy; 6 Department of Anesthesia and Intensive Care Unit and Pain Therapy, Mater Dei Hospital, Bari, Italy; 7 Intensive Care Unit, Department of Anesthesia, S. Maria Goretti Hospital, Latina, Italy

**Keywords:** Fascial plane blocks, Locoregional anesthesia, Cardiac surgery, ERAS, Pain management

## Abstract

In recent years, there has been a growing awareness of the limitations and risks associated with the overreliance on opioids in various surgical procedures, including cardiothoracic surgery.

This shift on pain management toward reducing reliance on opioids, together with need to improve patient outcomes, alleviate suffering, gain early mobilization after surgery, reduce hospital stay, and improve patient satisfaction and functional recovery, has led to the development and widespread implementation of enhanced recovery after surgery (ERAS) protocols.

In this context, fascial plane blocks are emerging as part of a multimodal analgesic in cardiac surgery and as alternatives to conventional neuraxial blocks for thoracic surgery, and there is a growing body of evidence suggesting their effectiveness and safety in providing pain relief for these procedures.

In this review, we discuss the most common fascial plane block techniques used in the field of cardiothoracic surgery, offering a comprehensive overview of regional anesthesia techniques and presenting the latest evidence on the use of chest wall plane blocks specifically in this surgical setting.

## Introduction

Over the last decade, there has been a notable trend in surgical techniques and approaches toward less invasive procedures, and regional anesthesia has evolved to complement these changes [1]. In particular, new and more superficial regional anesthesia techniques have been developed to align with the principles of minimally invasive surgery and enhanced recovery protocols [2].

The shift toward opioid-sparing techniques and the incorporation of regional anesthesia into cardiothoracic surgery pain management protocols reflect a broader trend in medicine toward improving patient outcomes, reducing complications, and enhancing the overall surgical experience for the patient [1].

Enhanced recovery after surgery (ERAS) protocols [1, 2] aims to improve functional recovery and patient outcomes trough a multimodal multidisciplinary approach in order to minimize surgical stress response and postoperative pain, alleviate suffering, gain early mobilization after surgery, reduce hospital stay, and improve patient satisfaction.

In this context, regional anesthesia is a valuable component of ERAS protocols for cardiac and thoracic surgery [1, 2]. By providing effective pain relief while minimizing opioid use, it can contribute to improved patient outcomes and a faster recovery process.

Traditionally, opioids have been a cornerstone of pain management in cardiothoracic surgery.

In recent years, there has been a growing awareness of the limitations and risks associated with the overreliance on opioids in cardiothoracic surgery and other surgical procedures.

Today, there is a new generation of regional anesthesia techniques, called fascial plane blocks [3].

These blocks are emerging as an effective alternative to conventional techniques such as paravertebral, epidural, or spinal blocks, and involve the injection of a local anesthetic between the muscles through which the peripheral nerve travels. The nerve itself is not targeted, and the needle is not directed toward the neural axis; therefore, the risks of serious complications such as neural injury and neuraxial hematoma can be prevented or at least reduced [4].

In this review, we discuss the most common fascial plane blocks used in the field of cardiothoracic surgery, offering a comprehensive overview of regional anesthesia techniques and presenting the latest evidence on the use of chest wall plane blocks specifically in this surgical setting.

### Thoracic fascial plane blocks

Understanding the anatomy of fascial layers and their significance in regional anesthesia is crucial for anesthesiologists when administering nerve blocks and interfascial plane blocks to manage pain during various medical procedures.

Regarding fascial tissue anatomy in the human body, 3 fascial connective layers must be addressed [4]:

- Superficial fascia: this is the layer of connective tissue that lies just beneath the skin. It contains fat and provides a separation between the skin and underlying structures.
- Deep fascia: the deep fascia is a dense membrane of connective tissue that extends throughout the body. It surrounds and encases muscles, nerves, and other structures. It plays a crucial role in providing structural support and compartmentalizing different body regions.
- Muscle-related fascial layers: within the context of muscles, there are three primary fascial layers: epimysium (the connective tissue that surrounds the entire muscle); perimysium (which surrounds bundles of muscle fibers called fascicles); and endomysium, which surrounds individual muscle fibers within the fascicles.

The deep fascia is particularly important in the setting of interfascial plane blocks because it is the primary target for these blocks. Deep fascia is a continuous membrane that connects various anatomical structures, including mechanoreceptors and nervous fibers. Targeting the deep fascial layers with regional anesthesia techniques can effectively block sensory input and provide pain relief [4, 5].

Advances in ultrasound technology have made it easier to identify and target specific fascial planes within the thoracic region (Table 1). This has certainly contributed to the growth in popularity of these techniques, also allowing to abandon more risky blind approaches.

**Table 1:** Fascial plane blocks for cardiothoracic surgery and their indications

Block	Target	Indication	Highest level of evidence
SPIP/DPIP	Anterior cutaneous branches from T2 to T6 intercostal nerves	Median sternotomy S-ICD implantation (with SAPB)	Level I Level IV
PECS I–II	Medial and lateral pectoral nerves, lateral cutaneous branches of intercostal nerves (T2–T7), long thoracic and thoracodorsal nerves	Thoracotomy VATS surgery Median sternotomy Mini-invasive MV surgery S-ICD implantation Trans-subclavian TAVI	Level II Level IV Level II Level II Level IV Level V
SAPB	Lateral cutaneous branches of intercostal nerves (T3–T8), long thoracic and thoracodorsal nerves	VATS surgery Mini-invasive MV surgery S-IDC implantation	Level I Level II Level IV
ESPB	Ventral and dorsal ramus of the thoracic spinal nerve, lateral cutaneous branches of intercostal nerves (T2–T8)	VATS surgery and open thoracotomy Mini-invasive MV surgery	Level I Level II

*SPIP* Superficial parasternal intercostal plane block, *DPIP* Deep parasternal intercostal plane block, *SAPB* Serratus anterior plane block, *ESPB* Erector spinae plane block, *VATS* Video-assisted thoracic surgery, *MV Surgery* Mitral valve surgery, *S-IDC* Subcutaneous implantable cardioverter-defibrillator; level of evidence were assigned as defined by the Oxford Centre for Evidence-Based Medicine [6]

We can classify thoracic fascial plane blocks into:

- Anterior chest wall blocks: parasternal intercostal plane block (including superficial and deep parasternal block);
- Antero-lateral chest wall blocks: includes pectoral nerve block (pectoralis nerve block I and II) and serratus anterior plane block;
- Posterior chest wall block: includes the erector spinae plane block.

## Methods

An extensive literature search was conducted for the present review. For this, a computer-based search was performed in PubMed, Embase, and Scopus databases. The date range for the search was set from January 2010 to September 2023.

After reading the titles and abstracts, the records obtained from the database were pre-selected by the authors. Those whose, after reading the full text, were not thought to be consistent with the review in terms of content were excluded. Since our study was designed as an article in the category of narrative review, standard methodological methods and statistical analysis were not used in meta-analyses.

We evaluated the quality of data available on each plane block and assigned a level of evidence as previously defined by the Oxford Centre for Evidence-Based Medicine (Table 1) [6].

### Parasternal block—parasternal intercostal plane block

The parasternal intercostal plane (PIP) block targets the anterior cutaneous branches from T2 to T6 intercostal nerves, covering the anterior and medial chest wall. The technique can be divided into superficial and deep PIP block depending on whether the local anesthetic (LA) is deposited above or below the intercostal muscle (IM). Due to the nervous overlap, a bilateral injection is required to ensure the full coverage of the sternal area. PIP block provides analgesia and anesthesia after median sternotomy, sternal fractures or subcutaneous implantable cardioverter defibrillator (S-ICD) implantation, and to the internal mammary region after artery harvesting [7–9].

A further indication is breast surgery of the medial region of the chest or radical mastectomies in association with the interpectoral plane (PECS I), pectoserratus plane (PECS II), and serratus anterior plane block (SAPB) [10–12].

### Superficial PIP block

The superficial PIP block was first introduced by Torre et al., in 2014, under the name of pectointercostal fascial plane block, and then redefined by Fusco et al., in 2017, as an analgesic technique used in breast surgery to cover the medial region of the mammary gland [13, 14], and in combination with the PECS I, PECS II, and SAP blocks.

**Fig. 1:**
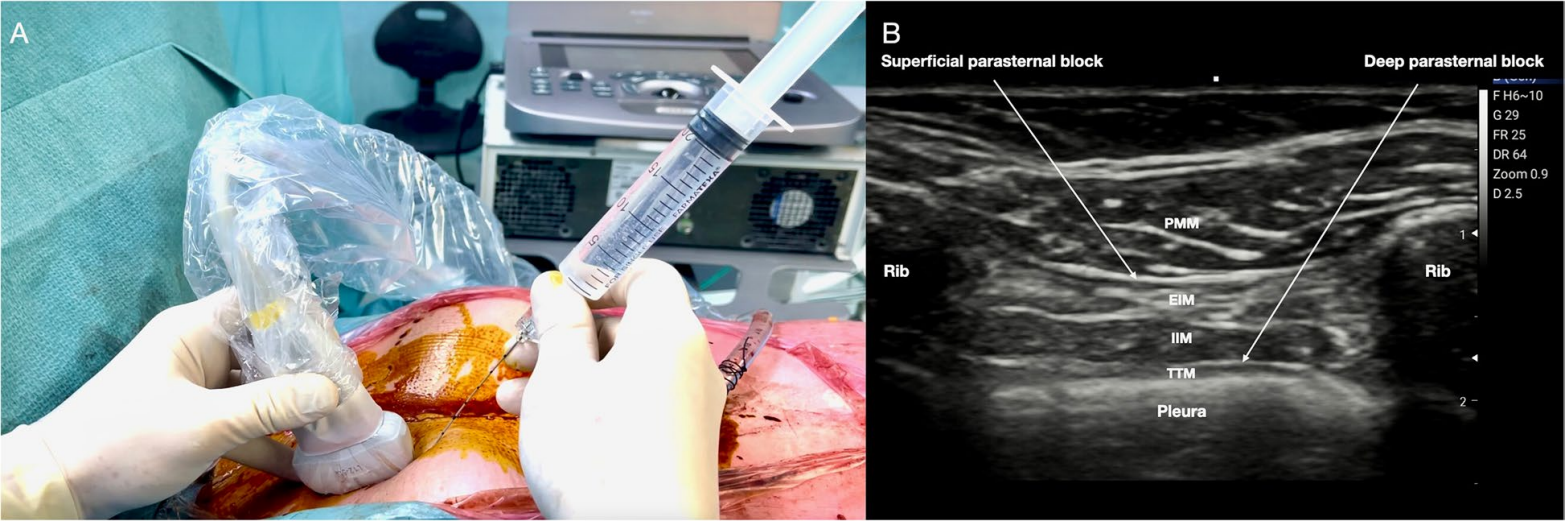
**A** Superficial parasternal block. Patient and probe position. The needle is inserted with the in-plane approach in the cranial to caudal or caudal to cranial direction. **B** Ultrasound landmarks of superficial and deep parasternal block. From top to bottom: pectoralis major muscle, external intercostal muscle, internal intercostal muscle, transversus thoracis muscle, pleura, ribs. PMM, pectoralis major muscle; EIM, external intercostal muscle; IIM, internal intercostal muscle; TTM, transversus thoracis muscle

The superficial PIP block is performed with the patient in the supine position. A high-frequency linear probe is positioned parallel and laterally to the sternum at the level of the second and fourth intercostal space. The needle is inserted with the in-plane (IP) approach in the cranial to caudal or caudal to cranial direction into the fascial plane between the pectoralis major muscle (PMM) and the external intercostal muscle (EIM) (Fig. 1A, B).

**Fig. 2:**
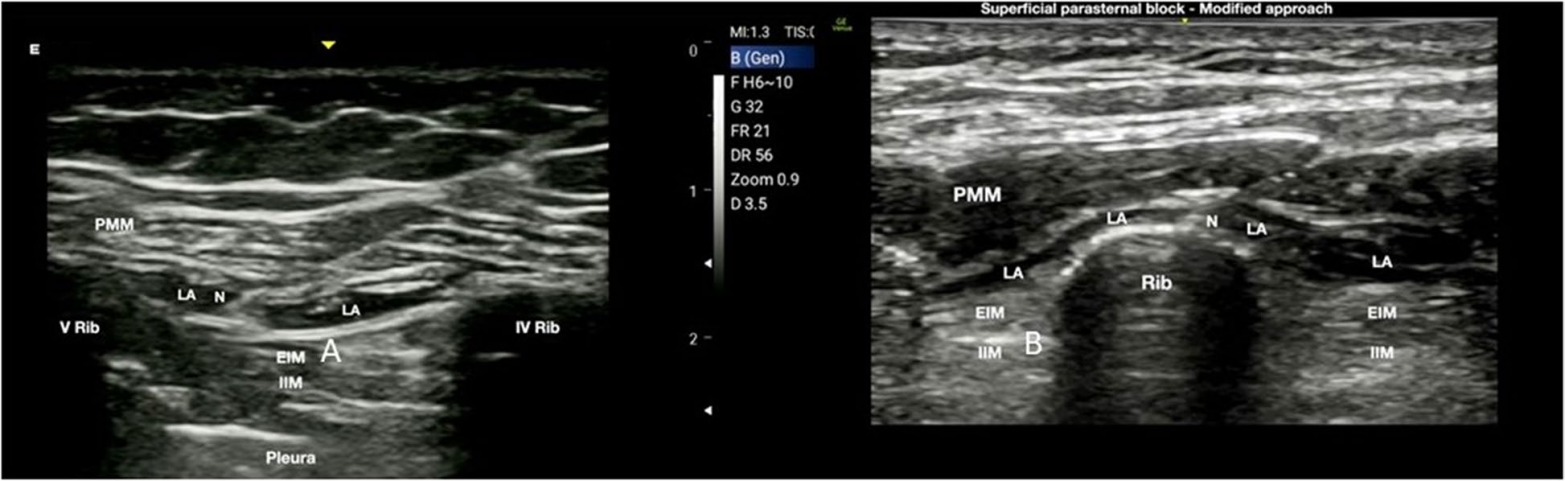
**A** Superficial parasternal block. At level of second and fourth intercostal spaces, the local anesthetic is injected between the pectoralis major muscle and the external intercostal muscles. PMM, pectoralis major muscle; EIM, external intercostal muscle; N, needle; LA, local anesthetic. **B** Superficial parasternal block–modified approach. The injection on the dome of the rib allows a more homogeneous and longitudinal diffusion of local anesthetic, obtaining a better dermatomal coverage. PMM, pectoralis major muscle; IIM, internal intercostal muscle; EIM, external intercostal muscle; N, needle; LA, local anesthetic

Some clinicians have observed that the anatomical convexity of the ribs might limit the correct and adequate diffusion of the volume of LA in the fascial plane of the intercostal space, hindering fascial hydrodissection, and reducing the dermatomal spread (Fig. 2A).

As a result, Sepolvere et al. have suggested a modified approach by positioning the tip of the needle on the rib dome in order to reduce the local anesthetic volume and obtain a more homogeneous and longitudinal spread into the target fascial compartment [15] (Fig. 2B).

Initially proposed as an analgesic technique to manage intra- and postoperative pain from cardiac sternotomy, the superficial PIP block has been also described in case reports as an anesthetic procedure for sternal surgery (debridement, total resynthesis) on patients in spontaneous breathing with high comorbidities, considered at risk of serious pulmonary complications related to general anesthesia and intubation [16–18].

### Deep PIP block or transversus thoracis plane block

First described in 2015 by Ueshima H et al. as the transversus thoracis plane (TTP) block [19], the deep PIP targets the deep anterior cutaneous nerves, which run into the anatomical plane between the TTM and the PMM.

The patient and probe position, such as the approach and the needle insertion, are the same for both superficial and deep block (Fig. 1A). The ultrasound landmarks of deep PIP are represented in Fig. 1B. The LA is injected between the internal intercostal muscle (IIM) and the TTM.

**Fig. 3:**
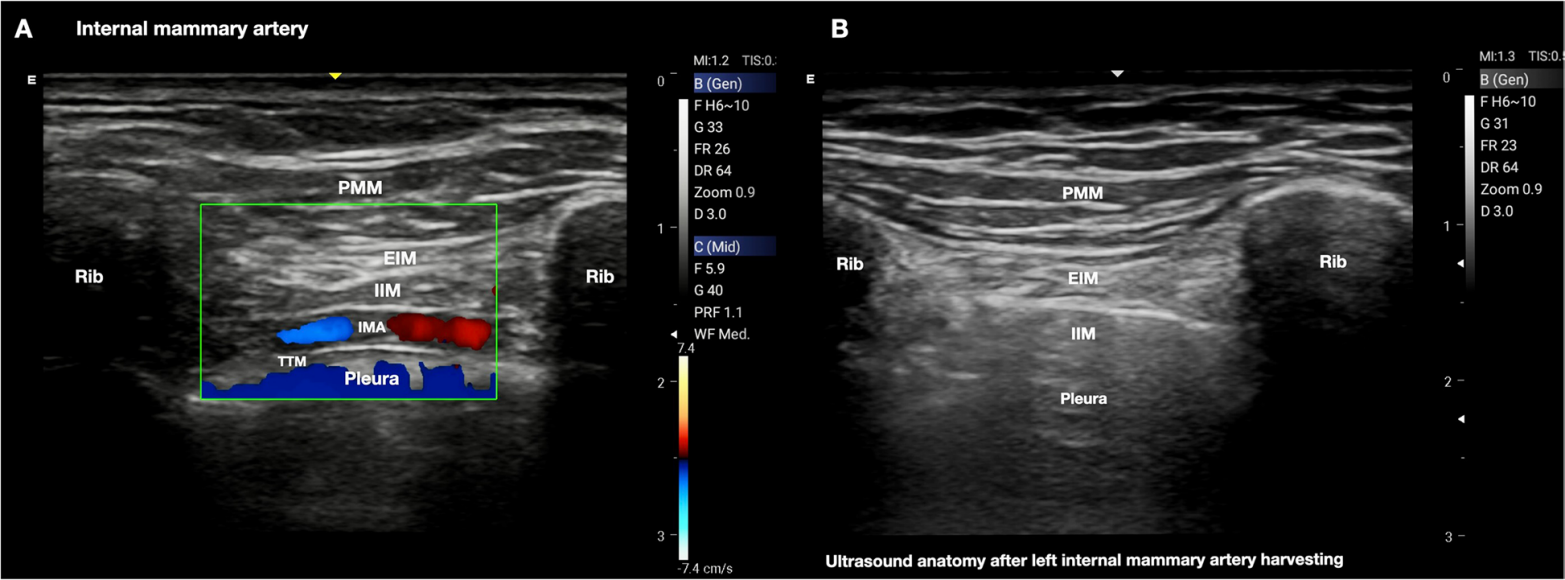
**A** The internal mammary artery can be identified between the internal intercostal and transversus thoracis muscles. This anatomical plane represents the target of the transversus thoracis plane block. PMM, pectoralis major muscle; EIM, external intercostal muscle; IIM, internal intercostal muscle; TTM, transversus thoracis muscle; IMA, internal mammary artery. **B** Ultrasound anatomy after left internal mammary artery harvesting. Patients can have tissue disruption in the transversus thoracis plane muscle after the internal mammary artery harvest, making transversus thoracis muscle identification more difficult, and the deep parasternal intercostal plane block almost impossible to perform. PMM, pectoralis major muscle; EIM, external intercostal muscle; IIM, internal intercostal muscle

Both superficial and deep PIP block are effective in blocking the anterior cutaneous branches of the thoracic intercostal nerves (Th2–6) [13], but some significant anatomical considerations are warranted. The TTM is a very tiny and variable muscle in many people and difficult to visualize with ultrasound. At the level of the fourth parasternal rib intercostal space, the internal mammary artery (IMA) and internal mammary vein (IMV) can be identified between the IIM and TTM as a longitudinal pulsatile structure approximately 1.5 cm from the lateral border of the sternum, making the fascial target of TTP block the same anatomical plane in which both IMA and IMV run **(**Fig. 3A).

On this basis, the risk of pneumothorax, local anesthetic systemic toxicity (LAST), and IMA injury or hematoma should be considered when a deep PIP block is performed. Moreover, patients can have tissue disruption in the transversus thoracis plane after IMA harvesting, making TTM identification more difficult, and the deep PIP block almost impossible to realize (Fig. 3B).

Furthermore, if the block is done before cardiac surgery, both the right and left IMAs could be damaged, rendering the arteries unusable for bypass grafting. If the block is performed after left IMA harvesting at the end of coronary artery bypass grafting (CABG), only the right IMA can be damaged. Some believe that the superficial PIP block is a more protective and safer technique for IMA compared to deep PIP block performed far from vascular structures, pleura, and heart [20].

### Literature review

The analgesic efficacy of deep PIP block after cardiac surgery has been reported in several trials in terms of satisfaction and opioid sparing, suggesting its potential role in fast track cardiac anesthesia [21–24].

Li et al. [25], in a meta-analysis involving 12 randomized controlled trials (RCTs) with a total of 730 patients, aimed to assess the impact of parasternal block (PSB), including both superficial and deep blocks, on various outcomes in the field of cardiac surgery and postoperative pain management. Patients in the PSB group reported lower numerical rating scale (NRS) scores compared to the control group at specific time points, including at extubation, 4 h after surgery, and 8 h. This indicates that PSB was associated with better pain relief during the immediate postoperative period. The use of PSB was found to reduce the incidence of postoperative nausea and vomiting (PONV). Moreover, patients who received PSB had a significantly reduced mechanical ventilation time, shorter lengths of stay in the ICU, and overall hospital stay.

In line with these results, King et al. [26], in a meta-analysis, assessed the effect of PSB in patients undergoing cardiac surgery with a sternotomy approach. In their analysis, they included several approaches, including superficial and deep PIP and both single-shot or continuous infusion. They reported a significant reduction in postoperative pain and opioid use.

In another meta-analysis, Liu et al. explored the adverse effects of deep PIP, observing that the procedure provides effective postoperative analgesia and inconclusive incidence of PONV, though no further complications or side effects were considered [27].

In a cadaveric study, Samerchua et al. evaluated the optimal injection technique of both superficial and deep PIP to cover the T2–T6 intercostal nerves, the presence of TTM and its ultrasound identification, and the location of the IMA. They found that TTM were found at the second and up to sixth intercostal spaces, and the IMA was located along the second to sixth intercostal spaces medially to the halfway point between the sternal border and the costochondral junction. Moreover, the dye spread after superficial PIP block was better and more localized compared to the deep PIP block. They concluded that an optimal technique for superficial PIP block was a triple injection at the second, fourth, and fifth intercostal spaces, while a double injection at the third and fifth intercostal spaces represented the best technique for the deep PIP block [28].

In another cadaveric model, Harbell et al. [29] opted for a deep PIP injection between rib 3 and 4 and observed consistent spread to 4–6 intercostal levels.

A current and controversial opinion in cardiac surgery is whether the PIP block should be performed before or at the end of surgery. Padala et al., in a single-blind, randomized trial compared the preincisional and postincisional PIP block, concluding that both the choices provide equivalent pain relief during the postoperative period [30]. Despite extubation time was significantly higher in the preoperative group, the authors identified higher duration of surgery and cardiopulmonary bypass as possible source of bias.

Zou et al. reported, in a double-blind, randomized trial, that the preemptive deep PIP block reduced the intraoperative opioid need and provided effective analgesia during the first 12 postoperative hours in patients undergoing off-pump CABG [31].

### Rectus sheath block

The rectus sheath block (RSB) provides somatic analgesia to the anteromedial abdominal wall and periumbilical area after midline laparotomy abdominal surgery, targeting the anterior cutaneous branches from the T9 to T12 intercostal nerves. First described as a blind technique in 1899 by Schleich, the introduction of ultrasound made the ultrasound guided RSB a safe and effective ERAS procedure, reducing the opioid requirements and avoiding many complications related to neuraxial techniques and vascular injury.

In addition to the pain after median sternotomy, drainage catheter insertion sites are highly painful locations after cardiac surgery and, therefore, an adequate tube site pain control has an important role in overall pain management after cardiac surgery. Positioning is burdened by a high incidence of severe postoperative pain reported in the epigastric region, impairing the patient’s respiratory dynamics, potentially leading to prolonged weaning from ventilation [32, 33].

**Fig. 4:**
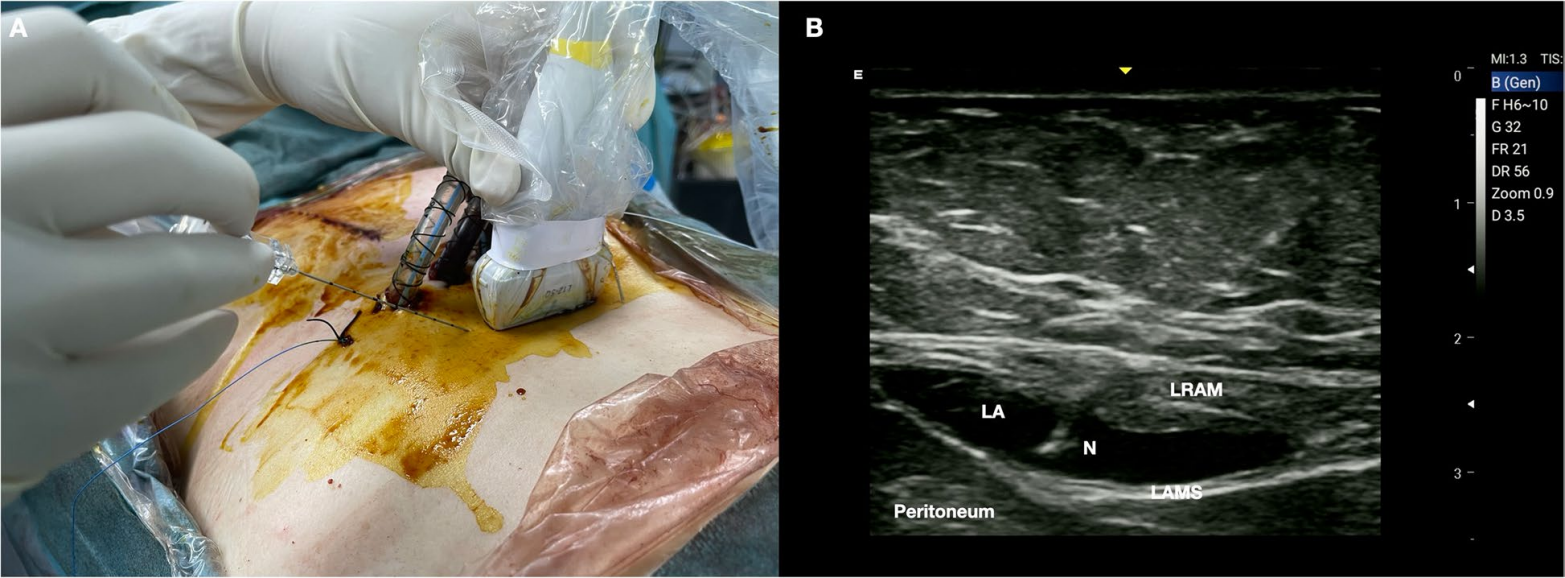
**A** Patient and probe position. The patient is in the supine position and a high-frequency linear probe is placed just below the tube emergence from the skin. **B** The local anesthetic injected between the rectus abdominis muscle and its sheath. LRAM, left rectus abdominis muscle; LAMS, left abdominis muscle sheath; N, needle

RSB can be performed at the end of the surgery after subxiphoid drainage placement. The patient is in the supine position, and a high−frequency linear probe is positioned just below the tubes emerging from the skin (Fig. 4A). The ultrasound landmarks are represented by the skin, the right (RAM) and left (LAM) rectus abdominis muscles, the rectus abdominis muscles sheath (RAMS), and the peritoneum.

Through an IP approach and a bilateral injection (lateral to medial first and medial to lateral subsequently), 15–20 ml of LA for each side is deposited between the rectus muscle and its sheath (Fig. 4B)

### Literature review

RSB has a potential role as part of effective multimodal opioid-sparing approach in combination with superficial or deep PIP both to manage pain deriving from median sternotomy and subxiphoid drainages, as reported in two recent case series in the pediatric and adult populations [34, 35].

Toscano et al., in a case report, described the combined use of PIP and RSB blocks, also known as pectoralis-intercostal rectus sheath (PIRS) plane block, for pain in a fragile patient undergoing mediastinal revision via subxiphoid access due to deep sternal wound infection [36].

This technique can also be performed at induction of general anesthesia through the introduction of bilateral dwelling catheters that run from the epigastrium, where a single shot RSB is first administered bilaterally, to the sternal notch covering T1 to T10 dermatomes through a continuous infusion of LA in patients undergoing cardiac surgery via sternotomy, and receiving subxiphoid drainages [37, 38].

A further potential and innovative application for pain management after left ventricular assist device (LVAD) implantation via median sternotomy is the combination of bilateral deep PIP block to provide analgesia to the sternal region and unilateral RSB to ensure pain relief to left abdominal structures through which the LVAD driveline was inserted. This approach was described in a case report, resulting in good quality analgesia and leading to a quick discharge from the ICU without need for rescue analgesia administration [39].

### Pectoralis nerve blocks

The PECS block is a fascial plane block that provides analgesia to the upper anterior chest wall. The PECS 1 block targets the medial and lateral pectoral nerves, anesthetizing the pectoralis muscles. The PECS 2 block, an extension of the PECS 1 block, provides an additional injection to block the upper intercostal nerves (T2–T7, including the intercostobrachial nerve) and, in addition, the long thoracic and thoracodorsal nerves. Due to their relative efficacy, simplicity, and safety, the PECS blocks have quickly gained in popularity [40]. Blanco first described the PECS 1 block for providing analgesia after breast surgery involving pectoralis muscles, and later described the PECS 2 as a modification of the PECS 1 for more extensive surgeries and those involving axillary dissections [11, 12]. Recently, in order to standardize the nomenclature of regional anesthesia techniques, a consensus was drafted by representatives of both the American Society of Regional Anesthesia and Pain Medicine (ASRA) and the European Society of Regional Anesthesia and Pain Therapy (ESRA), referring to the PECS I block as an interpectoral plane block, and PECS 2 as pectoserratus plane block [41].

**Fig. 5:**
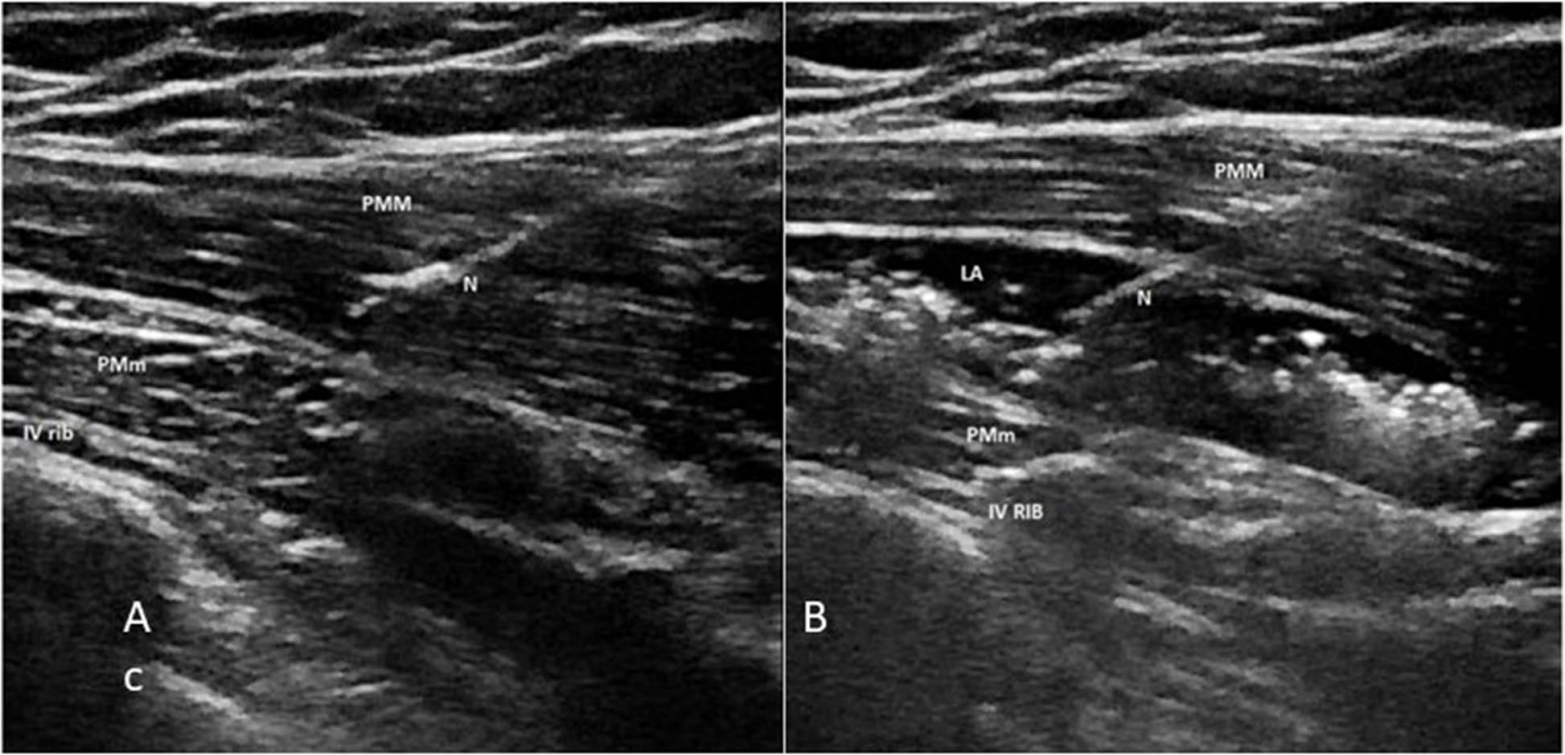
**A** PECS 1 before injection of local anesthetic. **B** PECS 1 after injection of local anesthetic, which spreads into the fascial layers between pectoral major and minor muscles. PMM, pectoralis major muscle; PMm, pectoralis minor muscle; N, needle; LA, local anesthetic

The PECS 1 block can be carried out using a linear ultrasound probe. The probe is positioned in a sagittal orientation, in the deltopectoral groove. When the axillary artery and vein are visualized, the probe is slid downwards until ribs are visualized (Fig. 5A). Pectoralis major is recognized as the large superficial muscle beneath the subcutaneous tissue, with the pectoralis minor deeper into it. At this level, there is the pectoral branch of the thoracoacromial artery, which pulses between the pectoralis muscles, with the lateral branch of the pectoral nerve typically close to the artery. Usually, a volume of 10 ml of LA is deposited in the fascial plane between the pectoralis muscles to perform a standard block [11, 41] (Fig. 5B).

**Fig. 6:**
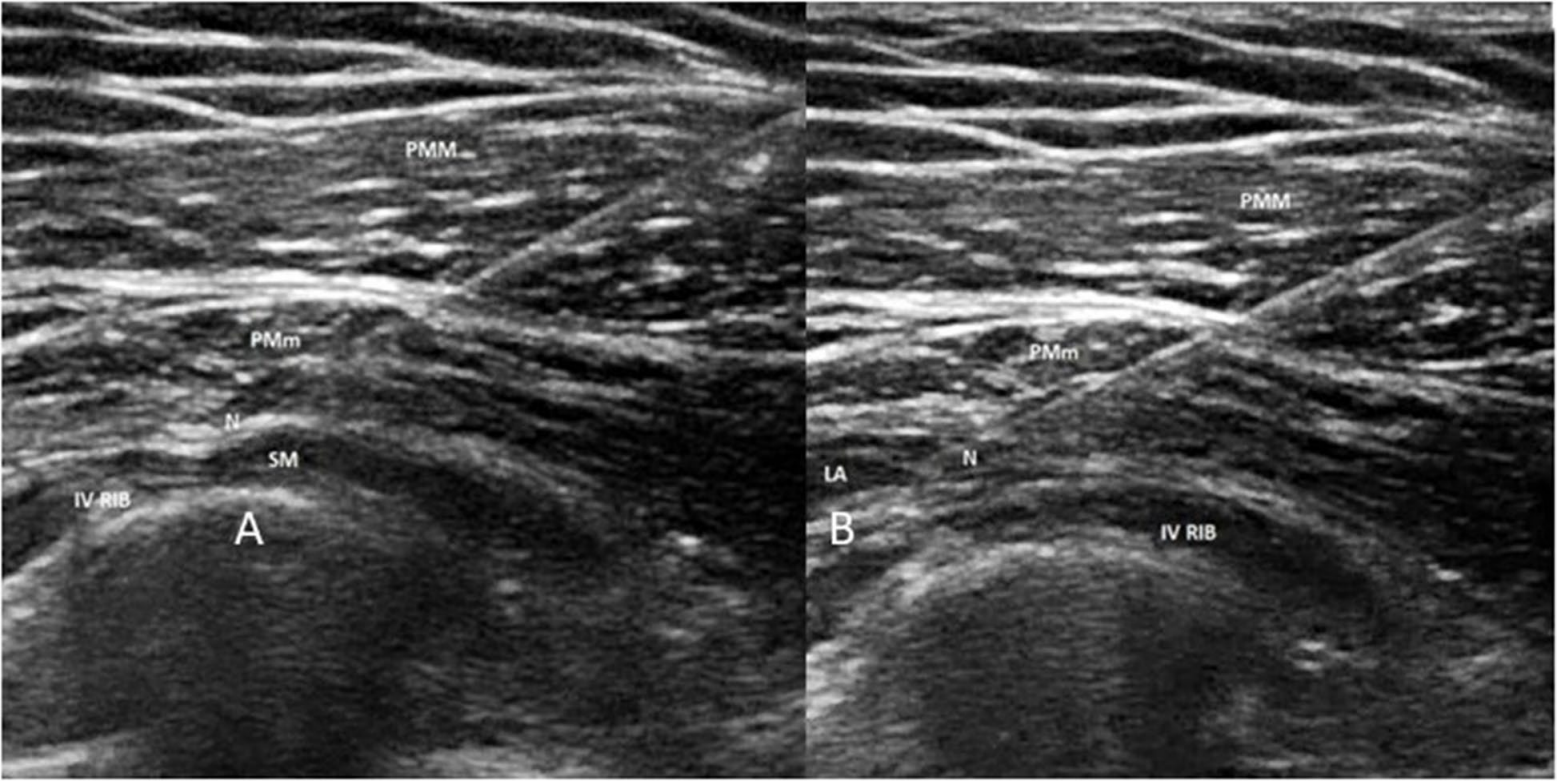
**A** PECS 2 before injection of local anesthetic. **B** PECS 2 after injection of local anesthetic, which spreads into the fascial layers between pectoral minor and serratus anterior muscles. PMM, pectoralis major muscle; PMm, pectoralis minor muscle; SM, serratus anterior muscle; N, needle; LA, local anesthetic

PECS 2 is the modified version of the PECS 1 block and consists of two separate injections. The first injection aims to deposit LA in the fascial plane between pectoralis muscles (same as PECS 1 block). Then, sliding the probe onto the anterior axillary line, the 3rd and 4th ribs are visualized (Fig. 6A). A slight rotation is made to the probe to allow needle insertion along a superiormedial to inferior-lateral passage. Usually, 20 ml of LA is then deposited in the plane between pectoralis minor and serratus anterior muscles [12, 41] (Fig. 6B).

### Literature review

First described by Blanco for providing analgesia after breast surgery, PECS blocks have progressively been used for a wide variety of breast surgery procedures. In 2020, the PROSPECT Working Group (a collaboration of surgeons and anaesthetists working to formulate procedure-specific recommendations for pain management in several surgical settings) developed recommendations based on a procedure-specific systematic review of randomized controlled trials. According to PROSPECT recommendations for oncological breast surgery, PECS blocks were suggested for major breast surgery if no axillary node dissection is performed or if paravertebral block (PVB) is contraindicated (grade A recommendation) [42]. However, a review of the same recommendation made by Italian opinion leaders has highlighted that PECS 2 block can be used to cover muscles, axilla, and lateral branches of the intercostal nerves (T2–T7), allowing surgeries involving the axilla (level 1), such as sentinel node biopsy and axillary dissection. For this reason, when the axilla is involved, PECS 2 block represents an effective and safer alternative compared to PVB, which is not an “entry level” block due to its potential difficulties and complications. Moreover, PVB does not cover the cervical and brachial plexus branches, which contribute to the innervation of the breast and axillary region [43–45]. PECS blocks have also demonstrated its analgesic efficacy in plastic surgery [46]. However, a promising field of application is cardiothoracic surgery, including thoracotomy, video-assisted thoracoscopic surgery (VATS), median sternotomy, cardiac interventional procedures, thoracic trauma, and Port-a-Cath insertion [47].

Kumar et al. [48] randomized 40 patients scheduled for CABG or valve surgeries via midline sternotomy to postop PECS block or no block. In the PECS group, extubation was significantly earlier, with a median ventilatory duration of 108.5 ± 24.33 versus 206.3 ± 47 min and pain scores at rest and with cough were also significantly lower at zero, three, six, 12, and 18 h after surgery.

Moreover, Marcoe et al. [49] compared 112 patients who received PECS 1 block, subcostal transversus abdominis plane (TAP) block multimodal analgesia, or multimodal analgesia without regional block. The group that received regional blocks had a 51.1% reduction in intraoperative opioid requirements (*p* < 0.001).

Yalamuri et al. [50] also described a case in which PECS 2 block was used as rescue analgesia in a patient undergoing mitral valve repair via right anterior thoracotomy. However, despite potentially useful, no trials are available for the use of PECS in mini invasive cardiac surgery.

In a prospective randomized trial, Yildirim et al. [51] compared thoracic paravertebral block (TPVB) with PECS II for postoperative analgesia in patients undergoing VATS. They enrolled 26 patients for each group and no significant differences were found in static and dynamic VAS score in the first 24 postoperative hours. However postoperative morphine consumption and rescue analgesia requirement were higher in the PECS group. On the other side, PECS II block provided better intraoperative hemodynamic stability compared to TPVB. In 2021, Luo et al. [52] recruited 40 adult patients undergoing lobectomy, segmentectomy, and partial lung resection and highlighted how PECS 2 block preconditioning significantly reduced surgical stress, stabilized blood flow, and prolonged the time required for first analgesia. It also reduced surgical pain related to the incision, and opioid consumption, often responsible for serious side effects.

PECS blocks have also been successfully used as a valid analgesic technique in pediatric surgery.

Kaushal et al. [53] compared the effectiveness of ultrasound-guided deep SAP block, PECS 2 block, and intercostal nerve block in the treatment of pain after thoracotomy in pediatric cardiac surgery. These blocks showed comparable effectiveness on pain scores in the early postoperative period (1 to 4 hours).

Kamal et al. [54] compared the analgesic effect of bilateral PECS 2 block with conventional intravenous analgesia (control) on post-sternotomy pain after cardiac surgery in children. The block group had lower pain scores and lower postoperative opioid requirements compared with the control group. Additionally, agitation upon awakening and length of stay in the ICU were lower in the study group.

The mechanism by which PECS 2 blockade mediates post-sternotomy analgesia is unclear. Though these blocks are not expected to cover the anterior cutaneous branches of the intercostal nerves, several studies have reported effective analgesia with PECS blocks during median sternotomy in pediatric cardiac surgery [48, 54]. In fact, for median sternotomy, bilateral PECS 2 blockade has been shown to be superior to systemic analgesia alone, though the mechanism is unclear. PECS blocks are not expected to block the anterior cutaneous branches of the intercostal nerves; however, they may exert an analgesic effect by reducing the spasm in pectoral or intercostal muscles [47].

Pectoral nerve blocks have also been successfully used in interventional cardiac procedures. PECS 2 alone and in combination with general anesthesia has been shown to facilitate cardiac device implantation and transcatheter procedures [55], while PECS 1 block for cardiac device implantation in combination with general anesthesia has been shown to provide optimal analgesia in children [56]. PECS blocks have also been used with success for Port-a-Cath insertion [57].

### Serratus anterior plane block

The SAPB is a fascial plane block, and initially described by Blanco et al. [58] with the intent of providing analgesia for the lateral chest. It is an appropriate choice for procedures involving the lateral thorax (anterolateral thoracotomy, robotically assisted approaches).

The SAPB can be performed with two different techniques, depending on the LA injection site: in the superficial SAPB, the LA is deposited in the interfascial plane between the serratus anterior muscle (SAM) and the latissimus dorsi muscle; in the deep SAPB, the LA is deposited between the SAM and intercostal muscles at the intersection of the V rib.

**Fig. 7:**
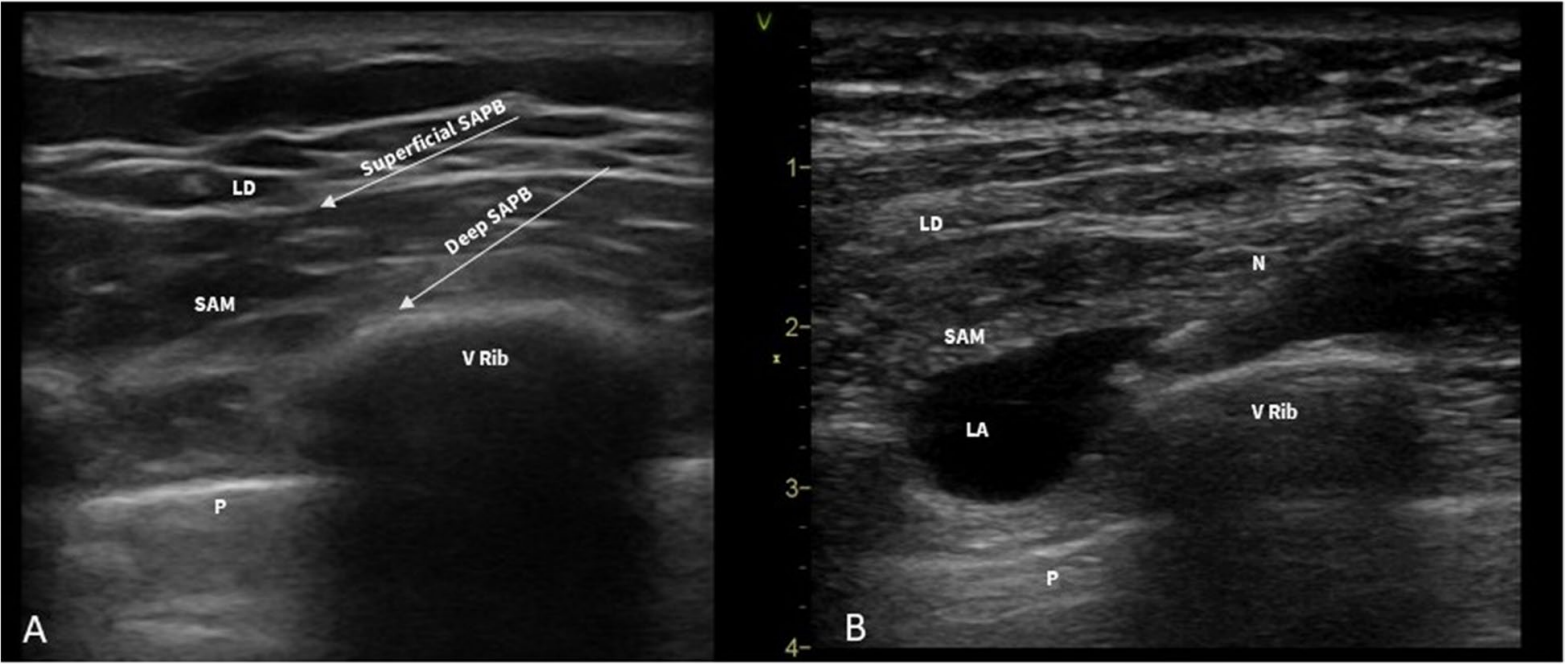
**A** Sono-anatomy of SAPB: In the superficial SAPB, the target is the interfascial plane between LD and SAM; in the deep SAPB, the target is the interfascial plane between SAM and the periosteum of the V rib. **B** Deep SAPB: the needle is advanced in-plane in the interfascial plane between the SAM and the V rib and LA is injected. LD, latissimus dorsi; SAM, serratus anterior muscle; P, pleura; LA, local anesthetic; N, needle

SAPB can be performed as single-shot or continuous block through the placement of a peripheral nerve catheter in the interfascial plane. With the patient in lateral or supine position, an ultrasound probe is placed at the level of the midclavicular line in a sagittal plane. The ribs are counted with caudal and lateral probe movement until the fourth and fifth ribs are identified in the midaxillary line. At this level, the SAM, visible as a small hypoechoic muscle, overlies the ribs, while the latissimus dorsi muscle is clearly identifiable superficial to the SAM (Fig. 7A).

The needle is introduced in the caudal-cephalad direction using an in-plane approach, and after confirming the position of the tip by hydrodissection, LA is deposited above (superficial SAPB) or below (deep SAPB) the SAM (Fig. 7B).

**Fig. 8:**
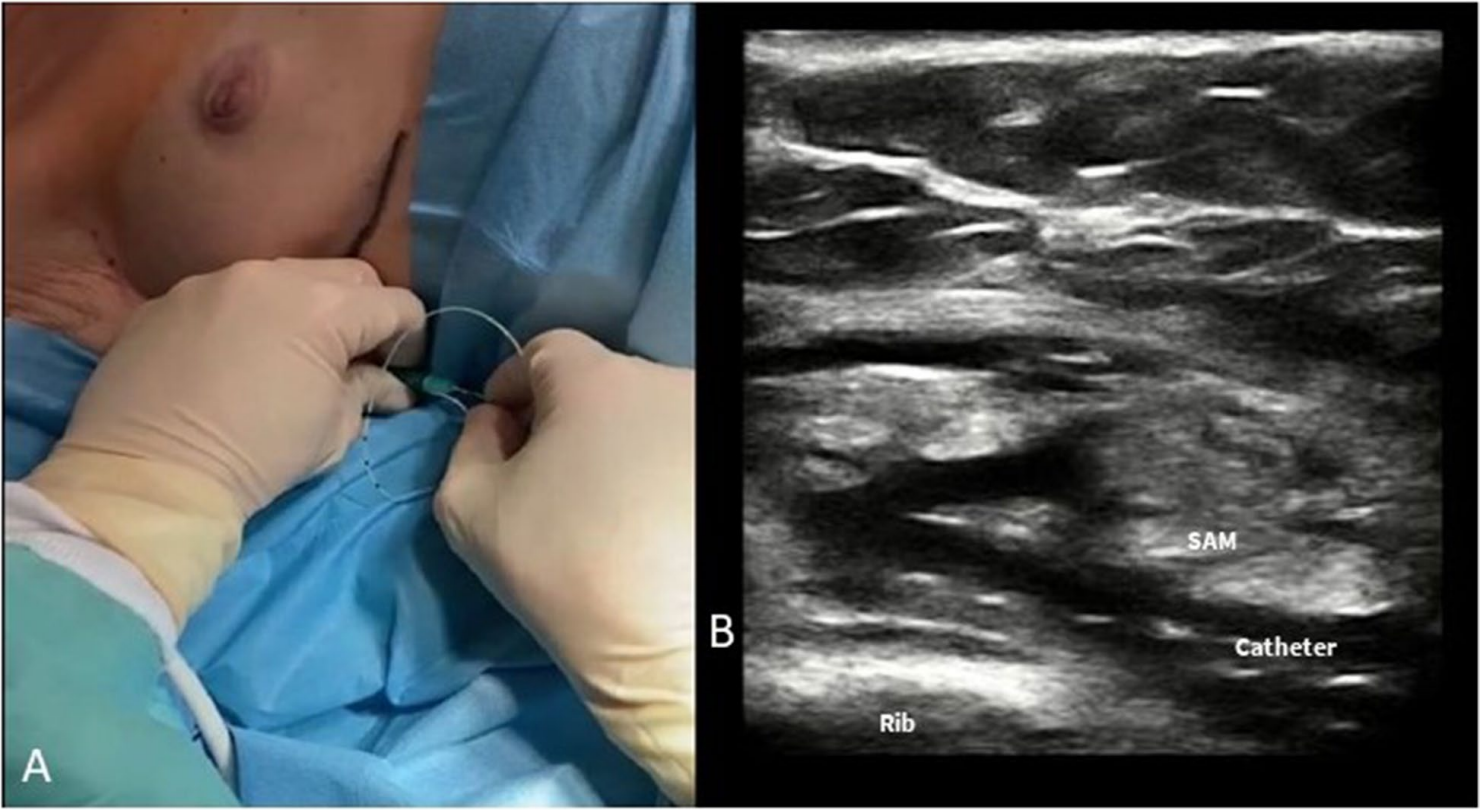
**A** Placement of the SAPB catheter with the patient in supine position. **B** Ultrasound visualization of the SAPB catheter between the SAM and the rib. SAM, serratus anterior muscle

After the interfascial space is opened with LA or saline, a catheter can be placed inside the serratus anterior muscle plane with a continuous infusion of LA (continuous SAPB). Deep SAPB may be preferable in authors opinion because the deeper approach can provide simplified sonographic imaging, and by inserting the catheter between the serratus muscle and V rib, there is probably less risk of dislocation [59] (Fig. 8A, B).

The lateral cutaneous branches of the intercostal nerves traverse both the deep and superficial serratus anterior planes and are the targets of both blocks. At the midaxillary line, the lateral cutaneous nerves penetrate the external intercostal and serratus anterior muscles, further dividing into anterior and posterior divisions, which innervate the anterolateral chest wall.

Daga et al. [60] undertook a descriptive cadaveric study to delineate the extent of the cephalo-caudal spread of the injectate after superficial SAPB, showing the diffusion area, including the lateral cutaneous branches of the second to sixth intercostal nerves, the long thoracic nerve, and the thoracodorsal nerve. Therefore, anatomically, both superficial and deep SAPB can block the lateral cutaneous branch of the intercostal nerve, while superficial SAPB also affects the long thoracic and the thoracodorsal nerves that lie on top of the SAM.

Blanco et al. demonstrated a longer duration and better spread of LA with the superficial block compared to the deeper [58]. However, two human cadaveric studies examined the spread of dye after 20 ml or 40 ml of methylene blue injection: increasing the volume from 20 to 40 ml doubled the area of injectate spread and promoted dye spread preferentially to the anterior chest wall and axilla, providing a more reliable analgesic coverage; consequently, using 40 ml of solution, the duration and distribution were the same for the superficial and the deep block, precisely from T2 to T9 [61, 62].

### Literature review

Initially utilized in breast surgery [63], the use of SAPB has greatly increased in both thoracic and cardiac surgery. The guidelines for the perioperative management of patients undergoing lung surgery recommend SAPB in single-port VATS or when paravertebral blockade is not appropriate (e.g., pleurectomy and decortication) [2].

Despite the small incision, a moderate-severe postoperative pain following VATS is not rare, affecting expectoration and leading to complications, such as pulmonary infection [64]. Viti et al. randomly allocated 94 patients eligible for VATS into two groups: opioid-based systemic pain treatment (control group) and the same systemic analgesics regimen plus pre-emptive SAPB (interventional group). They found that SAPB provided better pain control and, consequently, a better performance during postoperative rehabilitation exercises in terms of duration and quality of incentive spirometry [65].

SAPB represents a safe, effective alternative to standard of care in the treatment of thoracic pain in the acute setting, such as rib fractures [66] or chest tube placement [67].

Continuous SAPB has been described in case reports and case series on post-operative pain management after lung transplantation [68] for post-thoracotomy analgesia [69] and for pain relief in multiple fractured ribs [70]. Moreover, SAPB has been successfully used for the treatment of post-thoracotomy [71] and postmastectomy pain [72]. SAPB has also achieved a prominent role in postoperative pain management in minimally invasive cardiac surgery via thoracotomy.

An observational cohort study evaluated the analgesic impact of continuous deep SAPB (interventional group *n* = 33) against opioid-based systemic analgesia (control group *n* = 26) for patients undergoing minimally invasive mitral valve surgery through right minithoracotomy. The authors noted a marked reduction in morphine consumption in the first 24 h in the interventional group, allowing good quality analgesia with low consumption of opioids [59].

In a double-blind RCT, Gautam et al. achieved similar results, showing that SAPB reduced the postoperative pain scores and opioid requirements in patients undergoing minimally invasive direct coronary artery bypass (MIDCAB) surgery [73].

Moreover, SAPB, alone or combined with parasternal block, has been shown to be an effective and durable means of treating pain associated with subcutaneous implantable cardiac defibrillator (S-ICD) placement [74].

SAPB has been compared to erector spinae plane block in also in thoracic surgery With regard to thoracotomy, ESPB has shown a superior analgesic profile to SAPB in several clinical trials [75, 76]. Specifically, Elsabeeny et al. [75] compared, in a randomized trial, the analgesic efficacy of ESPB, SAPB, and TEA in perioperative pain control in thoracotomy surgery. While ESPB and TEA showed a nearly comparable analgesic profile, both were superior compared to SAPB. The postoperative morphine consumption was also higher in patients in the SAPB group.

Taking all of this into account, the authors suggested a possible role of ESPB as alternative to TEA in thoracotomy surgery, with a secondary role for the SAPB. Mixed results were instead found for VATS: Ekinci et al. [77] found lower pain scores and lower opioid consumption in the ESPB group compared with deep SAPB; in a pilot randomized comparison trial, Gaballah et al. [78] found comparable results, while Finnerty et al. [79] showed the superiority of ESPB, both in analgesic effect and opioid consumption.

In line with these studies, the PROSPECT guidelines for VATS cannot actually consider SAPB as first‐line treatment until its efficacy compared with the more established paravertebral and ESP blocks has been confirmed by other studies [80].

However, the same PROSPECT guidelines underlined how the SAPB, which is simple and quick to perform with limited side-effects, could bring benefits in terms of pain and opioid consumption compared with systemic basic analgesia. This is also confirmed by a recent meta-analysis by De Cassai et al. [81], which assessed the analgesic efficacy of SAPB compared with general anesthesia only for VATS peri-operative pain control. In their study, seven RCTs, for a total of 489 patients, were included. In the SAPB group, pain scores at 6, 12, and 24 h were reduced, together with a reduction in postoperative opioids (mean difference − 4.81 mg of intravenous morphine equivalent, 95% CI − 8.41 to − 1.22) and in the incidence of nausea and vomiting.

### Erector spinae plane block

The ESPB was first described by Forero, in 2016, and is considered a relatively recent addition to the field of regional anesthesia and pain management [82]. The ESPB involves the injection of LA into the interfascial plane located between the erector spinae muscles and the tip of the transverse vertebral process.

**Fig. 9:**
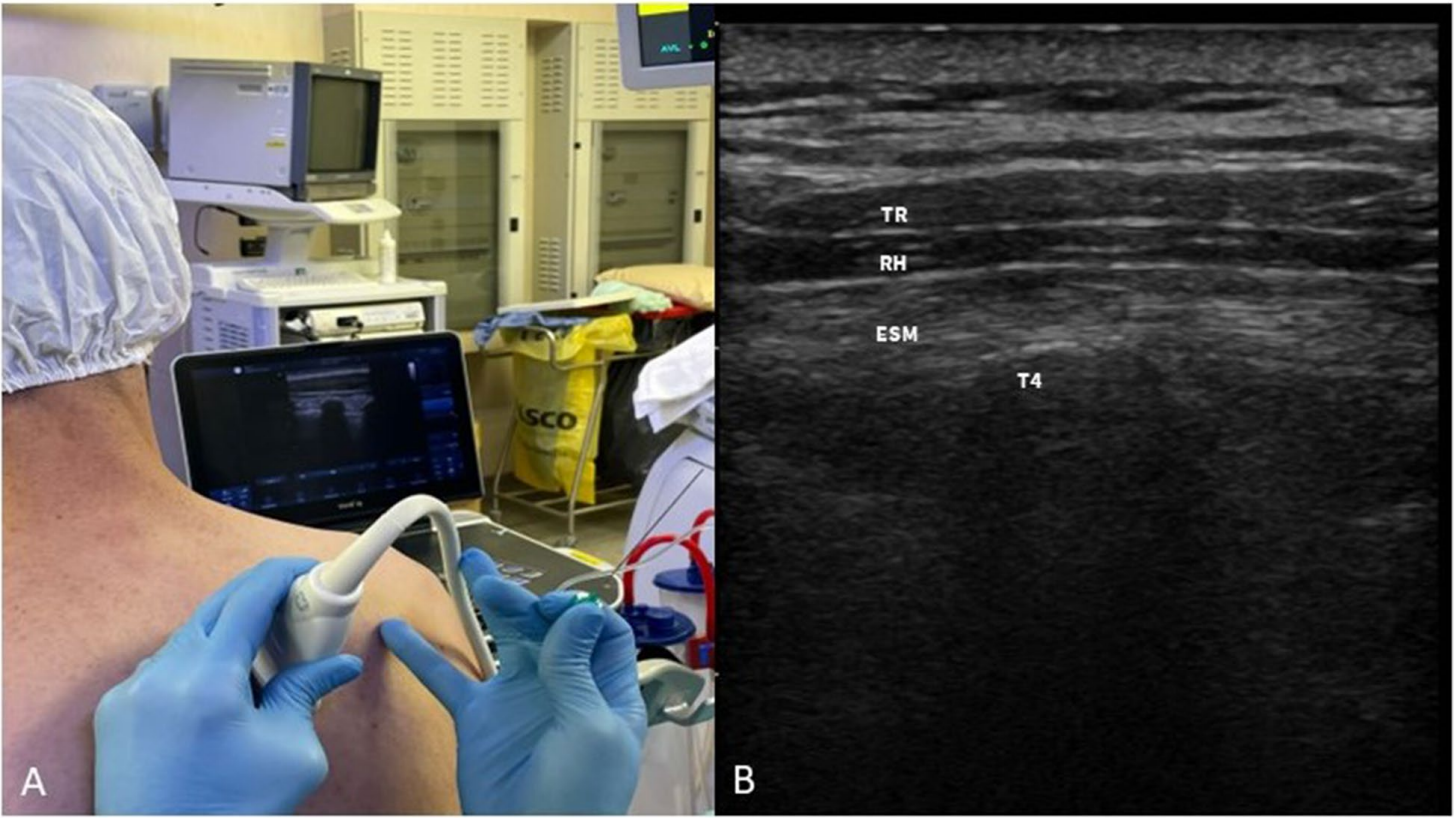
**A** ESPB performed with the patient in sitting position before general anesthesia induction. **B** Sono-anatomy of the ESPB block: trapezius (uppermost), rhomboid (middle), and erector spinae (lowermost) are identified. The hyperechoic T4 transverse process is individuated inferior to the erector spinae muscle. TR, trapezius muscle; RH, rhomboid muscle; ESM, erector spinae muscle; T4, transverse process

ESPB can be performed in different positions, depending on the clinical situation and the preferences and expertise of the health care provider. ESPB can be performed with the patient in a sitting position (Fig. 9A), prior to general anesthesia, or can also be performed with the patient in a lateral position. This can be done either before or after the induction of general anesthesia, depending on the clinical circumstances and the anesthesia team’s preferences.

To perform the block in cardiothoracic surgery, a linear probe is generally placed in a sagittal view at the level of T4-spinous process, 2–3 cm laterally from the midline in order to identify the T4 transverse process, the trapezius, rhomboideus, and erector spinae muscles (Fig. 9B).

**Fig. 10:**
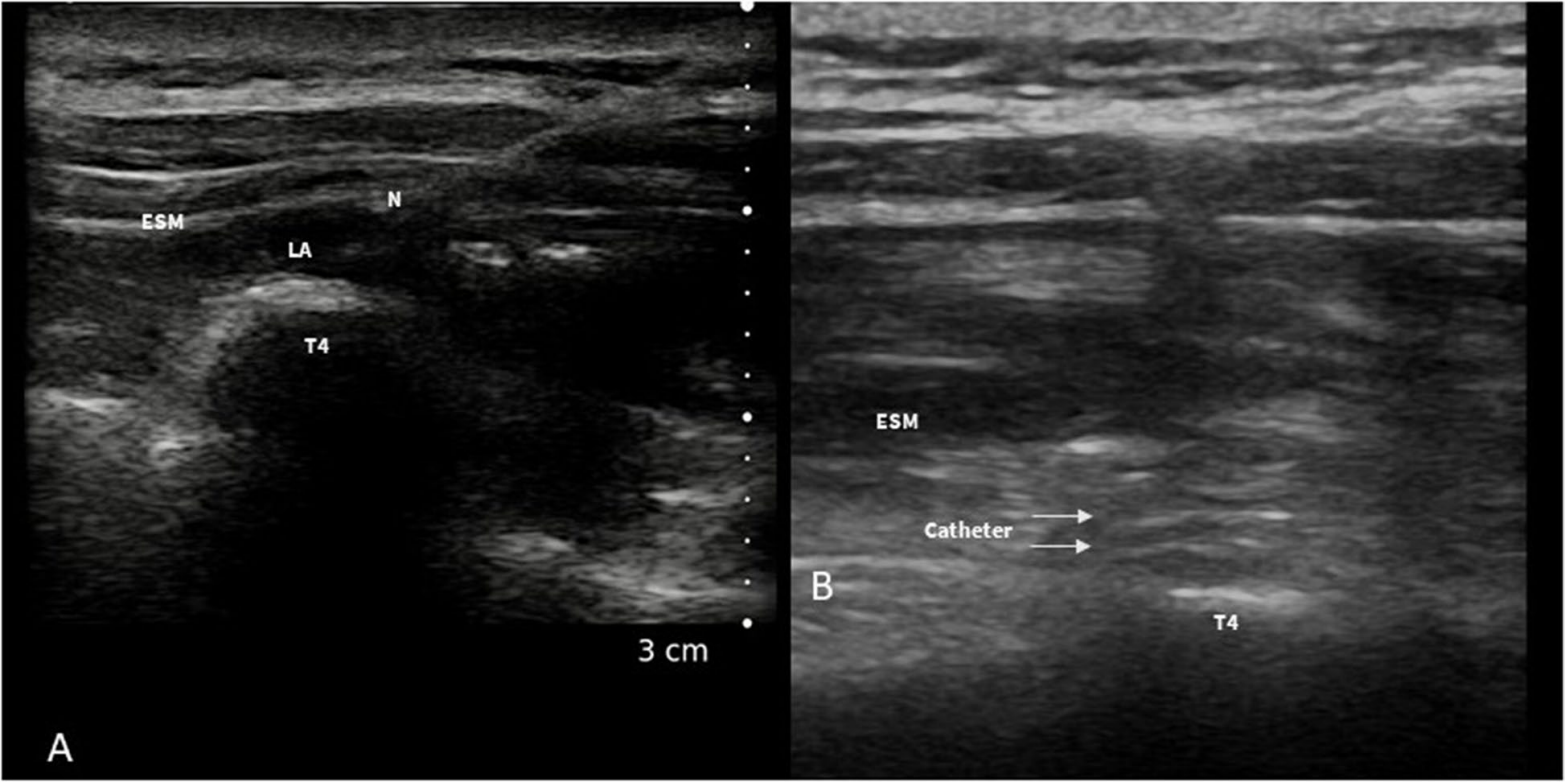
**A** ESPB: the needle is advanced with an in-plane approach in caudo-cranial direction to reach the interfascial plane between the transverse process and the erector spinae muscle. **B** Ultrasound visualization of the ESPB catheter between the ESM and the transverse process. ESM, erector spinae muscle; LA, local anesthetic; N, needle; T4, transverse process

The needle is then inserted via an in-plane approach, and LA is injected in the caudal to cranial direction into the interfascial plane between the erector spinae muscle and the transverse process (Fig. 10A). By the placement of a peripheral nerve catheter in the interfascial plane, a continuous ESPB can be performed (Fig. 10B).

Since its first description, ESPB has gained attention and stimulated an interesting debate over its mechanism of action [83]. To date, thanks to several cadaveric studies and to magnetic resonance imaging, we know that ESPB has been found to provide both somatic (related to the body wall and musculoskeletal structures) and visceral (related to internal organs) analgesia. This suggests that it can be effective for managing pain originating from different sources [84, 85].

In fact, it has been observed that the LA injected into the fascial plane between the erector spinae muscles and the transverse vertebral processes can spread through channels in the intertransverse connective tissues, reaching the ventral and dorsal ramus of the thoracic spinal nerve along with the sympathetic ramus communicans at the intervertebral foramen level. Moreover, the involvement of lateral cutaneous branches of intercostal nerves contributes to the block’s analgesic effect.

Scharztmann et al. [84], using magnetic resonance imaging (MRI), have provided valuable insights into the spread and clinical effects of the ESPB injectate. The MRI study demonstrated that the injectate used in ESPB consistently spreads to several anatomical areas. These areas included the erector spinae muscles (which are the target of the block), neural foramina (openings through which spinal nerves exit the spinal column), and the intercostal space (the space between the ribs).

The distribution of the erector spinae muscle (ESM) from the neck to the lumbar region makes ESPB a versatile regional anesthesia technique. Its ability to provide analgesia in a wide area of the torso, including the thoracic region, has indeed contributed to its popularity in various surgical procedures. Here are just some of the reasons that ESPB has gained popularity, especially in cardiothoracic surgery.

### Literature review

Several randomized trials have been conducted to compare ESPB with other regional anesthesia techniques like epidural anesthesia, paravertebral blockade, and SAPB in thoracic surgery. ESPB is being actively studied and considered as a potential anesthesia technique in thoracic surgery, particularly for VATS and open thoracic surgery.

At present, enhanced recovery after surgery (ERAS) guidelines [2] recommend thoracic paravertebral block (TPVB) as a valid locoregional alternative to thoracic epidural anesthesia (TEA), with evidence of a better side effect profile. Therefore, in recent years, together with the growth in popularity of ESPB, several trials have compared it with PVB in thoracic surgery.

In a recent meta-analysis, which included 10 randomized trials on the topic, for a total of 624 patients, it was found that PVB was associated with improved pain scores only at 12 h after thoracic surgery, but with a lower opioid consumption at 48 h. On the other hand, ESPB was associated with a borderline significant trend toward reduction of block-related complications. These results suggest a possible role of ESPB as first choice, especially in patients undergoing VATS surgery and in those with coagulation disorders [86].

Regarding thoracotomy surgery, in a randomized trial, Das et al. [87] compared the analgesic efficacy and safety of preoperative, single-shot ultrasound-guided TPVB, ESPB, and SAPB in 90 patients scheduled for thoracic surgery undergoing posterolateral thoracotomy. They reported a better analgesic efficacy of ESPB compared with TPVB and SAPB, together with lower total opioid dose required and VAS score during the first 24 h.

Similar results were obtained in the already mentioned trial performed by Elsabeeny et al. [75], which compared ESPB, SAPB, and TEA for pain management in thoracotomy pain. Also in this trial, ESPB was superior to SAPB in terms of pain scores and opioid consumption in open thoracic surgery. In particular, outcome measures included 24 h postoperative visual analog scale (VAS), intraoperative rescue fentanyl consumption, and total postoperative morphine consumption. VAS scores at rest were significantly lower for the TEA group compared to the SAPB group and comparable between TEA and ESPB. In the SAPB group, 88.2% of the patients required postoperative morphine compared to 47.1% in the ESPB group and the total postoperative morphine consumption was higher in the SAPB group compared to the TEA and ESPB groups. Moreover, the first time to receive morphine was longer in the ESPB compared with SAPB group.

The efficacy of ESPB for VATS have been also described in several trials.

Zhao et al. [88], in a prospective randomized trial, compared ESPB with PVB for pain management in VATS surgery. The primary outcome was the postoperative oxycodone consumption at 48 h. Their trial showed the non-inferiority of ESPB regarding pain score, analgesic rescue consumption, and quality of recovery. Specifically, postoperative oxycodone consumption at 48 h was 7.9 ± 8.7 boluses in ESPB and 6.9 ± 6.3 boluses in PVB groups and the time to first oxycodone rescue was 16.1 ± 5.3 h in ESPB group vs. 15.8 ± 8.7 h in PVB group. No difference was detected in postoperative pain score at 12 h, 24 h, 36 h, and 48 h.

Finnerty et al. [79] compared ESPB and SAPB in terms of quality of recovery and overall morbidity after minimally invasive thoracic surgery. They found a clinically meaningful improvement in quality of recovery at 24 h for patients who received ESPB compared with a SAP block. Furthermore, ESPB showed a longer time to first opioid analgesia, and reduced pain at rest and on deep inspiration.

ESPB has also gained popularity as a valid alternative option for pain management in cardiac surgery. Athar et al. [89] compared bilateral ESB with 20 ml per side of 0.25% levobupivacaine (group E) or sham block with 20 ml of normal saline in patients undergoing elective on-pump single-vessel coronary artery bypass grafting or valve replacement. They found that single-shot ESPB decreased the first 24-h postoperative analgesic consumption by 64.5%, and risk of pain by five times in the patient population, also reducing the sedation and duration of mechanical ventilation in postcardiac surgery patients.

Similar results were reported in a randomized trial [90] that compared continuous ESPB with multimodal intravenous analgesia in 40 patients undergoing coronary bypass surgery. In this trial, the ESPB group showed a significantly lower VAS score than in the group of multimodal i.v. analgesia. Intraoperative fentanyl and postoperative morphine doses were significantly less in the ESPB group, together with shorter duration of mechanical ventilation.

In a randomized study, Nagaraja et al. [91] compared the effectiveness of ESPB catheters to thoracic epidural analgesia in adult patients undergoing median sternotomy for cardiac surgery.

Bilateral ESPB catheters were non-inferior to thoracic epidural analgesia at least for the initial 12 h post-extubation. However, beyond 12 h, the thoracic epidural group had improved pain scores. Importantly, there were no significant differences in the need for rescue analgesia, or other complications between the two groups.

Krishna et al. [92], in a prospective, randomized, single-blinded study involving adult patients undergoing cardiac surgery, compared the use of preinduction single-shot ESPB to a control group receiving paracetamol and tramadol. Patients in the ESPB group received a single-shot of ESPB with 3 mg/kg of 0.375% ropivacaine before surgery. Patients in the control group received 1 g of paracetamol every 6 h and 50 mg of tramadol every 8 h for pain management.

Patients in the ESP group had a significantly longer duration of analgesia, during which their pain score was less than 4 compared to the control group. The duration of effective pain relief in the ESPB group was reported as 8.98 ± 0.14 h, while it was 4.60 ± 0.12 h in the control group.

The efficacy of monolateral ESPB has been also described for minimally invasive cardiac surgery in several observational and retrospective trials [76, 93].

However, results from these studies are in contrast with a recent double blind randomized trial by Hoogma et al. [94] which compared 36 patients treated with continuous ESP with 36 control patients for minimally invasive mitral valve surgery. The authors did not found any significant differences in primary (cumulative morphine consumption during the first 24 h after extubation) and secondary outcomes (severity of pain, presence/extent of sensory block, duration of postoperative ventilation, and hospital length of stay).

The efficacy of ESPB in cardiac surgery has also been described in the pediatric population by Karacaer et al. [95], who reported a significant reduction in morphine consumption in the first 24 h using bilateral ESPB for postoperative pain management in children undergoing median sternotomy. Moreover, Macaire et al. described the use of bilateral ESPB catheters in a pediatric cardiac population, reporting reduced pain and opioid requirements [96].

The results described are in line with a recent metanalysis from Nair et al. [97]. In their analysis, the authors included 16 RCTs that compared ESPB with a control group (no block/sham block) in adult and pediatric patients undergoing cardiac surgeries for a total of 110 patients. The primary outcomes were postoperative opioid consumption and postoperative pain scores. They found that single-shot ESPB or continuous analgesia via catheter was associated with reduced intraoperative and 48 h opioid consumption and with reduced pain scores after extubation up to 16 h. Moreover, patients treated with ESPB had reduced duration of ventilation, reduced ICU and hospital stay, and early mobilization. However, no significant reduction in 24-h opioid consumption, PONV, and pruritus was observed in ESPB group compared with control. However, it must be noted that studies included in this metanalysis had a very high level of heterogeneity due to variable sample sizes, different surgical approaches and surgeries, and single-shot and continuous blocks.

### Safety of fascial plane blocks

Regional anesthesia techniques, including thoracic fascial plane blocks, can be associated with technical or needle-related complications. These may include bleeding at the injection site, inadvertent intravascular injection of local anesthetic, and nerve injury. In the thoracic region, where cardiac surgery often takes place, additional risks such as pneumothorax (air in the chest cavity) and hemothorax (blood in the chest cavity) should be considered.

Many patients undergoing cardiothoracic surgery may be on anticoagulation or antiplatelet therapy before, during, and after the procedure, potentially increasing the risk of bleeding complications associated with regional anesthesia techniques.

Evidence about the safety of fascial plane blocks in cardiac surgery is still sparse and the available literature consists mostly of case report and case series with a low level of evidence.

Adhikary et al. [98] reported the use of continuous ESPB for pain management in five patients undergoing left thoracotomy for left ventricular assist device placement. No complications were observed despite the need for prolonged postoperative heparinization.

Galacho et al. [99] reported no bleeding complications after ESPB in a case series of five patients with coagulation impairment (thrombocytopenia, INR > 1.5 and one patient on low-molecular-weight heparin anticoagulation).

A study by Toscano et al., in 2021 [100], provides valuable insights into the safety of fascial plane blocks, specifically continuous ESPB and SAPB in patients receiving anticoagulation and coagulopathy. The study involved a retrospective analysis of 70 patients undergoing minimally invasive mitral valve surgery through a right mini-thoracotomy. These patients received either continuous ESPB or SAPB for perioperative pain control, and coagulation status of patients at the time of catheter insertion and removal were assessed.

There were no adverse outcomes attributable to SAPB or ESPB in terms of vascular puncture, active bleeding, or hematoma formation, suggesting that these fascial plane blocks can be safely done in patients receiving anticoagulation and with coagulopathy.

Safety of fascial plane blocks have been also evaluated in thoracic surgery, with some data available from the analysis of secondary outcomes of RCTs.

Fang et al. [101], in a randomized trial, including 94 patients (not treated with antiplatelet or anticoagulant drugs) undergoing thoracotomy, reported 5 cases of hematoma in the group treated with PVB vs. 0 cases in the group treated with ESPB.

Similar results were reported by Chen et al. [102], in a randomized trial comparing intercostal nerve block, single-injection erector spinae plane block and multiple-injection paravertebral block on postoperative analgesia in thoracoscopic surgery. They reported 4 cases of hematoma in the PVB group (16.7% of a total of 24 patients) vs. 0 cases in the ESPB group.

Given the available evidence, it is not surprising that there are currently no specific guidelines available on the use of fascia blocks in patients considered at risk of bleeding.

Therefore, the safety of performing nerve blocks in patients on anticoagulant therapy is a subject of ongoing debate, and may vary depending on regional guidelines and expert opinions.

The American Society of Regional Anesthesia and Pain Medicine guidelines [103], regarding regional anesthesia in patients receiving antithrombotic therapy, recommend the need for caution in patients on anticoagulants or antiplatelet drugs. In cases where deep plexus or peripheral blocks are performed, the guidelines recommend applying neuraxial guidelines, which involve assessing the patient’s coagulation status and considering the risks of bleeding.

The Austrian Society of Anesthesiology, Resuscitation, and Intensive Care has a more permissive stance when it comes to performing superficial nerve blocks in patients on anticoagulants [104]. They do not consider systemic coagulation to be an absolute contraindication for certain nerve blocks.

A panel of Canadian experts on regional anesthesia reviewed the evidence and classified the risk of bleeding complications following peripheral regional nerve blocks [105]. They categorized the risk of bleeding complications for SAPB and PECS as intermediate, while ESPB was considered at low risk.

To conclude, although current literature suggests a greater profile of fascial plane blocks compared to neuraxial anesthesia, to date, we have no evidence that definitively confirms their safety in patients at high risk of bleeding, such as those on anticoagulant therapy or undergoing major cardiothoracic surgery.

Adequately powered randomized trial in the context cardiothoracic surgery are warranted to better determine their safety of TPVB and ESPB in patients with an increased risk of bleeding.

### Postoperative implications after loco-regional anesthesia: the fast track

The second millennium is the fast-track.

The concept of fast-track surgery, known today also as enhanced recovery after surgery (ERAS), was introduced in 1990 [106]. The goal was to optimize the perioperative care of surgical patients and accelerate their recovery while minimizing the stress response to surgery.

Since its first description, fast-track surgery has gained popularity in various surgical procedures including thoracic surgery, with the aim to improve patient satisfaction, reduce the length of hospital stays, and minimize the overall impact of surgery on patients’ daily lives.

The first, and pioneering, description of the fast track in thoracic surgery was that of Cerfolio et al. [107], which reported a case series of 500 pulmonary resection with a median discharge at day 4 (range 2–119 days) with 65% of the patients leaving before day 5, and a low 1.8% readmission rate, although they had a conventional thoracotomy. Cerfolio’s protocol included several elements intended to become the basis of the fast track, including early rehabilitation at day 1, epidural analgesia, removal of invasives by day 3, and discharge by day 4.

The implementation of VATS in thoracic surgery has facilitated the development of fast-track protocols or enhanced recovery after surgery (ERAS) programs in this field, and today, patients admitted for lobectomy are often discharged at home on the third postoperative day [108].

Achieving this outcome involves a comprehensive approach to perioperative management, with a specific focus on fast-track surgery principles, including prehabilitation and pulmonary rehabilitation programs, minimally invasive surgery approaches, an optimal intraoperative management and, obviously, a multimodal approach to pain [2].

Pain management is indeed a key component of fast track in thoracic surgery and is often a challenge for the anesthesiologist.

Pain after thoracic procedures can be debilitating and lead to poor outcomes, including respiratory complications such as atelectasis and pneumonia, as well as longer hospital stays, poor quality of life, and chronic persistent postoperative pain syndrome [109].

Together with recommendations for minimally invasive approach to surgery, ERAS strongly recommend a multimodal approach to pain, based on the combination of regional anesthesia techniques and non-opioid drugs, with the aim of reducing postoperative opioid use and its related side effects [2]. Actually, PVB is recommended as a valid alternative to TEA, with evidence of a better side effect profile and has become the first choice for postoperative analgesia in patients undergoing thoracic surgery, despite specific concerns, particularly regarding the risk of hematoma and pleural puncture, still remain.

Over the last 10 years, as surgery is moving toward less invasive techniques and approaches, regional anesthesia is also evolving and new and more superficial techniques have been described [3].

Specifically, in the context of thoracic surgery, new evidences about the efficacy of SAPB and ESPB are growing.

Capuano et al. [86], in a metanalysis, compared the analgesic efficacy of ESPB and PVB in thoracic surgery and reported a comparable pain scores at 6, 24, and 48 h after surgery, with a borderline significant trend toward reduction of block-related complications in favor of ESPB.

Similar considerations were advanced by Huang et al. [110] in another metanalysis, which showed that the PVB analgesic efficacy was comparable to that of ESPB in pain scores and opioid consumption at 24 h after surgery in a mixed population undergoing thoracic and breast surgery.

These findings are in line with the PROSPECT guidelines for VATS, which recommend the use of regional anesthesia techniques as fundamental components of fast track in thoracic surgery, suggesting the primary role of ESPB or PVB for postoperative pain management instead of the SAPB, considered as second choice [83].

Fast-track cardiac care is a complex intervention involving several components of care during cardiac anesthesia and in the postoperative period, with the ultimate aim of early extubation after surgery, and reduction of the length of stay in the ICU and in the hospital. Cardiac surgery can be associated with considerable postoperative discomfort and pain, and an optimal dynamic pain management has become a prerequisite for early postoperative recovery.

In the 1990s, high-dose opioid anesthetic strategies and mechanical ventilation in the ICU for 12 to 24 h were common [111]. Pain control based on the use of opioids was considered safe in hemodynamic terms and also protective for anti-ischemic and preconditioning effects [112]. This approach was eventually abandoned when it became well understood that the use and abuse of these drugs did not have beneficial results in these patients. On the contrary, it was the cause of a series of adverse effects (e.g., respiratory depression, bradycardia), ending in the extension of the extubation times. This idea was consolidated in 2019 with the enhanced recovery after cardiac surgery (ERACS) recommendations.

ERACS recommends effective perioperative pain control to improve patient outcomes, alleviate suffering, gain early mobilization after surgery, reduce hospital stay, and improve patient satisfaction and functional recovery [113]. In fact, poor pain management during the early postoperative period results in deleterious side effects on pulmonary (atelectasis, pneumonia, and bronchial secretion stasis), cardiovascular (increased oxygen consumption and tachycardia), and musculoskeletal (muscle weakness and disuse) systems that can prompt reintubation. As a solution, ERACS recommends multimodal analgesia, opioid sparing, and consideration of locoregional analgesia in all patients undergoing cardiac surgery procedures [112, 114].

Having now passed the era of neuraxial blocks (epidural and paravertebral block) due to the well-known side effects (e.g., epidural hematoma, pneumothorax) or due to concerns related to anticoagulation, cardiac anesthesia is oriented toward new regional techniques that can have the desired pain control as a final result. A number of studies have recently been published on fascial blocks in the cardiac surgery setting. The most studied blocks are parasternal superficial block, the pectoral block, and erector spinae plane block, and because these are all superficial blocks, they have fewer side effects and risk of hematoma formation [105].

A randomized control trial by Kumar et al., published in 2018 [48], analyzed forty adult patients undergoing coronary artery bypass grafting or valve surgeries through midline sternotomy. Group 1 patients did not receive PECS block, whereas group 2 patients received bilateral PECS block postoperatively. The PECS group patients required shorter duration of ventilator support (*p* < 0.0001) compared to the control group. Pain scores at rest and cough were significantly lower in the PECS group at 0, 3, 6, 12, and 18 h after extubation (*p* < 0.05). Furthermore, 34 episodes of rescue analgesia were registered in the control group, while in the PECS group there were only four episodes.

A more recent randomized control trial, by Zhang et al. [115], investigated the effect of bilateral pectointercostal fascial block in 108 patients undergoing open cardiac surgery allocated to receive either nerve block or no nerve block. This study concluded that the nerve block provides effective analgesia and accelerates recovery in terms of time of extubation, and ICU and hospital length of stay in patients undergoing open cardiac surgery.

A meta-analysis by Dost et al. [116] analyzed the effects of regional anesthesia on postoperative opioid consumption in patients undergoing open cardiac surgery. This study concluded that all the regional anesthesia techniques evaluated significantly reduced postoperative opioid consumption at 24 h, and that the ESPB was the most effective treatment.

A similar finding was published in another recent meta-analysis by Li J et al., in which the authors evaluated ultrasound-guided PSB, and found a decrease in opioid consumption, as well as improvement in clinical outcomes, such as mechanical ventilation time, total length of ICU stay, and hospital days [25].

Several fascial block techniques are currently utilized, and it is still challenging to determine which is better, given the lack of studies available in the literature. With this evidence, we can conclude that loco regional anesthesia is certainly a key pain control technique in this new era of cardiac surgery, as it spares use of opioids, reduces hemodynamic impact without interfering with the coagulative status of the patient, and enables a prompt fast track.

## Conclusion

Fascial plane blocks for cardiac and thoracic surgery represent the future of a well-established ERAS program. These blocks allow good quality analgesia with a simple and safe approach thanks to ultrasound guidance. However, the use of these blocks should be tailored to each patient’s needs, and future studies are needed to better understand their effects on patient outcomes.

## Data Availability

Not applicable.

## Abbreviations

ERAS: Enhanced recovery after surgery
PIP: Parasternal intercostal plane
LA: Local anesthetic
IM: Intercostal muscle
S-ICD: Subcutaneous implantable cardioverter defibrillator
PECS 1: Interpectoral plane
PECS II: Pectoserratus plane
SAPB: Serratus anterior plane block
IP: In-plane
PMM: Pectoralis major muscle
EIM: External intercostal muscle
TTP: Transversus thoracis plane
IIM: Internal intercostal muscle
IMA: Internal mammary artery
IMV: Internal mammary vein
LAST: Local anesthetic systemic toxicity
ICU: Intensive care unit
PSB: Parasternal block
RCTs: Randomized controlled trials
NRS: Numerical rating scale
PONV: Postoperative nausea and vomiting
CABG: Coronary artery bypass grafting
RSB: Rectus sheath block
RAM: Right rectus abdominis muscles
LAM: Left rectus abdominis muscles
RAMS: Rectus abdominis muscles sheath
PIRS: Pectoralis−intercostal rectus sheath
LVAD: Left ventricular assist device
ASRA: American Society of Regional Anesthesia and Pain Medicine
ESRA: European Society of Regional Anesthesia and Pain Therapy
VATS: Video−assisted thoracoscopic surgery
TAP: Transversus abdominis plane
SAM: Serratus anterior muscle
ESPB: Erector spinae plane block
MRI: Magnetic resonance imaging
ESM: Erector spinae muscle
TPVB: Thoracic paravertebral block
TEA: Thoracic epidural anesthesia
ERACS: Enhanced recovery after cardiac surgery
